# Novel Roles of GDF15 in Alleviating Renal Fibrosis: Promoting Autophagy and Lysosome Biogenesis via Inhibition of the PI3K/Akt/mTOR Pathway

**DOI:** 10.1111/jcmm.70951

**Published:** 2025-12-31

**Authors:** Youqi Li, Jinge Gu, Danping Tao, Haoyang Wu, Chen Yang, Yongjun Shi, Chengwen Huang, Boxun Luo, Jun Zhang

**Affiliations:** ^1^ Department of Nephrology Huizhou Central People's Hospital Huizhou Guangdong China; ^2^ Department of Nephrology, Zhujiang Hospital Southern Medical University Guangzhou China; ^3^ Department of Gerontology, Zhujiang Hospital Southern Medical University Guangzhou Guangdong China; ^4^ Department of Otolaryngology, Sun Yat‐Sen Memorial Hospital Sun Yat‐Sen University Guangzhou China; ^5^ Key Laboratory of Prevention and Management of Chronic Kidney Disease of Zhanjiang City, Institute of Nephrology Affiliated Hospital of Guangdong Medical University Zhanjiang Guangdong China

**Keywords:** autophagy, GDF15, macrophage infiltration, PI3K/Akt/mTOR pathway, tubulointerstitial fibrosis

## Abstract

Tubulointerstitial fibrosis (TIF) significantly contributes to the development of end‐stage renal disease (ESRD) in chronic kidney disease (CKD). However, the underlying mechanisms driving its development remain poorly understood, thereby impeding the development of effective prevention and treatment strategies. Although growth differentiation factor 15 (GDF15) has been implicated in kidney diseases, its specific relationship and mechanisms in the context of renal TIF remain unclear. In this study, we investigated the role and mechanisms of GDF15 in TIF using a mouse model of unilateral ureteral obstruction (UUO) and human tubular epithelial cells (HK2) stimulated by transforming growth factor‐β1 (TGF‐β1). Our findings demonstrated a downregulation of GDF15 expression in TIF. The upregulation of GDF15 mitigates renal TIF and reduces macrophage infiltration, whereas its downregulation exacerbates these conditions. Further analysis revealed that GDF15 promotes autophagy and lysosome biogenesis via the PI3K/Akt/mTOR signalling pathway, conferring a protective effect against TIF. In summary, our study demonstrated a negative correlation between GDF15 expression and renal TIF, highlighting its protective role in TIF. Moreover, GDF15 was found to promote autophagy and resolution of TIF through the PI3K/Akt/mTOR signalling pathway.

## 1 Introduction

Chronic kidney disease (CKD) poses a significant global public health challenge, with a prevalence ranging from 10% to 16% attributed to factors such as the rising incidence of hypertension, diabetes, and the aging of the population [1]. Tubulointerstitial fibrosis (TIF) is a common precursor to end‐stage renal disease (ESRD) in CKD of various etiologies, underscoring the crucial requirement for strategies aimed at preventing its progression [2]. Unfortunately, the regulatory mechanisms governing TIF remain elusive, posing a significant impediment to effective CKD management. Transforming growth factor‐β1 (TGF‐β1) plays a pivotal role in TIF progression towards ESRD [3, 4, 5, 6, 7]. However, direct targeting of TGF‐β1 has been shown to exacerbate renal damage by disrupting the protective effects of latent TGF‐β1 [8, 9]. This highlights the necessity of directing efforts towards downstream signalling molecules for TIF treatment. Growth differentiation factor 15 (GDF15), also known as macrophage inhibitory cytokine‐1, is a member of the TGF superfamily and functions as an autocrine regulator in macrophages [10]. GDF15 plays a role in organ growth, differentiation, development, and cellular repair, exhibiting anti‐inflammatory, anti‐proliferative, and anti‐tumour properties [11, 12]. Clinical investigations have demonstrated elevated serum GDF15 levels in CKD and other chronic inflammatory kidney conditions. Moreover, these studies have established a correlation between serum GDF15 levels and tubulointerstitial GDF15 mRNA, suggesting that GDF15 in the kidney may contribute to its presence in the bloodstream [13, 14, 15, 16, 17, 18]. GDF15 acts as an early mediator of the renal injury response, regulating inflammation, cell survival, proliferation, and apoptosis [19, 20]. Despite its association with the stress response, the precise biological role of GDF15 in renal interstitial fibrosis remains unclear. Thus, the present study aimed to investigate the potential role of GDF15 in renal fibrosis in patients with CKD and a mouse model of unilateral ureteral obstruction (UUO), while also delving into the mechanisms and therapeutic implications of GDF15.

## 2 Materials and Methods

### 2.1 Animal Experiment

C57BL/6J mice (male, 8‐week‐old) were purchased from the Guangdong Provincial Laboratory Animal Public Service Center and housed in specific pathogen‐free rooms. Each experimental group consisted of six to eight mice. The UUO operation was performed according to previously described methods [21, 22]. Briefly, the mice were anaesthetised via intraperitoneal injection. Next, a 1‐cm midline abdominal incision was created to mobilise and ligate the left ureter of the mice. In the sham operation group, the left ureter was mobilised without ligation, and the subsequent steps were identical. The mice were randomly allocated to ten distinct groups to examine GDF15 expression and its therapeutic potential in TIF: (1) sham operation group; (2) UUO group (Day 4); (3) UUO group (Day 7); (4) UUO group (Day 14); (5) sham operation + shNC group; (6) UUO (Day 7) + shNC group; (7) UUO (Day 7) + sh556 group; (8) sham operation + vector group; (9) UUO (Day 7) + vector group, and (10) UUO (Day 7) + GDF15 group. Kidney tissue samples were collected for immunohistochemistry, Western blot analysis, and real‐time PCR. All experimental procedures were approved by the Animal Ethics Committee of Zhujiang Hospital of Southern Medical University (LAEC‐2021‐224).

### 2.2 Cell Culture and Intervention

mTECs and HK2 cells were cultured as previously described. The mTECs and HK2 cells were serum‐starved overnight and treated with recombinant human TGF‐β1 (R&D Systems, USA) at a concentration of 10 ng/mL for 12, 24, and 48 h. To investigate the role of autophagy in TIF, we administered chloroquine at a concentration of 20 μM, followed by 48‐hour stimulation with TGF‐β1.

### 2.3 Plasmid Transfection

Cells were transfected with short hairpin RNA (shRNA) targeting GDF15 (sh865), negative control (shNC), GDF15 overexpression vectors, or empty vectors (GENECHEM, China) using Lipofectamine 3000 (Thermo Scientific, USA) once the confluence reached approximately 60%. Transfection procedures were performed according to the manufacturer's protocols. Twelve hours after transfection, the cells were exposed to 10 ng/mL of TGF‐β1 for 48 h. To investigate the effect of GDF15 on fibrosis in vivo, we initially screened shRNA in mTECs, with sh556 exhibiting approximately 70% downregulation, which was used in subsequent experiments. For tail vein injection of plasmids with ultrasonic microbubble guidance, a mixture of recombinant plasmid and the ultrasound contrast agent Sonovue (Bracco Diagnostics) in a 1:1 volume ratio was prepared 1 day before the surgery, as previously described [23]. Subsequently, 300 μL of the mixture (containing 150–200 μg of plasmid) was injected through the tail vein, and the mouse was immediately transferred to the device for ultrasound of the left kidney with an output power of 1 MHz and 1 W for 5 min.

### 2.4 siRNA Transfection

To investigate the role of GFRAL in GDF15‐mediated autophagy and fibrosis, we designed siRNA sequences specifically targeting the GFRAL gene. The sequences were generated using the Invitrogen Block‐iT RNAi Designer, and siRNAs with high silencing efficiency were selected (Table S1). These siRNAs were transfected into HK2 cells to verify gene silencing. Transfections were performed using Lipofectamine 3000 reagent, and after 48–72 h, total protein was extracted using RIPA lysis buffer. The expression level of GFRAL protein was subsequently assessed by Western blot analysis.

### 2.5 Bioinformatics Analysis

Genes identified as differentially expressed in the sequencing data were utilised for subsequent bioinformatics analysis. Bioinformatics analysis was performed using OECloud tools (https://cloud.oebiotech.cn). Venn diagrams were employed for cross‐analysing various datasets. To analyse the expression profile GSE23338 in the Gene Expression Omnibus (GEO) database, we used the GEO2R online analysis tool. The criteria for screening significant differences in upregulated and downregulated genes were defined as FC > 2.0 and *p* < 0.05. For pathway enrichment analysis, the Gene Ontology (GO) and Kyoto Encyclopedia of Genes and Genomes (KEGG) datasets were used. GO analysis of target genes was performed across three categories: biological process (BP), cellular component (CC), and molecular function (MF). Concurrently, KEGG pathway analysis was applied to analyse the key regulatory pathways. The screening and mapping of differentially expressed genes were primarily performed using online tools (https://cloud.oebiotech.cn).

### 2.6 RNA Extraction and Real‐Time PCR

Total RNA was extracted from kidney tissues and cultured cells using a TRIzol kit (GA, Japan) according to the manufacturer's instructions. Real‐time PCR was performed using the iQ SYBR Green Supermix with Opticon (Bio‐Rad, Hercules, CA, USA). The primers used are listed in Table S2. Relative mRNA expression levels were analysed using the comparative quantification method (ΔΔCt method) and normalised to GAPDH as the internal reference gene.

### 2.7 Masson's Trichrome, Picrosirius Red, and Immunohistochemical Staining

We performed Masson's trichrome staining using a Trichrome staining kit, following the manufacturer's instructions. Similarly, Picrosirius red staining was performed according to the manufacturer's instructions. Briefly, paraffin sections were dewaxed, rehydrated, and immersed in Mayer's haematoxylin for 30 s, followed by incubation with the Picrosirius red stain for 1 h. Immunohistochemical staining was performed according to a standard protocol. Briefly, paraffin sections were dewaxed, rehydrated, and subjected to antigen retrieval and inactivation of endogenous peroxidase. After blocking non‐specific antigen binding with goat serum, the sections were incubated overnight at 4°C with primary antibodies. Paraffin sections were subjected to immunohistochemistry using a microwave‐based antigen retrieval technique. The following antibodies were used: anti‐GDF15 (1:100; ab206414; Abcam), anti‐COL1A (1:100; 72026S; Cell Signalling Technology), anti‐FN (1:200; ab2413; Abcam), and anti‐LAMP1 (1:200; TD7033; Abmart). Next, the sections were incubated with HRP‐conjugated anti‐rabbit/mouse antibodies for 1 h at 37°C. Images were captured using a digital image analysis system and a microscope (Nikon, Japan). Positive signals were quantitatively analysed using an image analysis system (AxioVision 4; Carl Zeiss, Jena, Germany), as previously described.

### 2.8 Immunofluorescence Co‐Localisation Staining

Immunofluorescence staining was performed on paraffin‐embedded kidney tissues collected from UUO model mice on postoperative day 7 and sham‐operated control mice. After deparaffinisation, rehydration, antigen retrieval, and blocking, tissue sections were incubated with primary antibodies overnight at 4°C. To determine the localisation of GDF15 and its receptor GFRAL within the nephron, co‐staining was conducted using segment‐specific tubular and glomerular markers. The primary antibodies used were as follows: anti‐GDF15 (rabbit polyclonal antibody; 1:200; bs‐3818R; Bioss), anti‐GFRAL (rabbit monoclonal antibody; 1:100; ab315899, Abcam), anti‐AGTR1 (rabbit polyclonal antibody; 1:100; ab124505; Abcam), anti‐NCC (rabbit polyclonal antibody;1:100; ab95302; Abcam), anti‐AQP2 (rabbit monoclonal antibody; 1:200; ab199975; Abcam), LTL lectin (FITC‐conjugated *Lotus Tetragonolobus* Lectin; FL‐1321‐2; Vector Labs), and anti‐WT1 (rabbit monoclonal antibody;1:200; ab89901; Abcam).

To avoid cross‐reactivity resulting from the use of primary antibodies raised in the same host species (e.g., GDF15 with AGTR1, GDF15 with AQP2, or GFRAL with NCC), a tyramide signal amplification (TSA) system (Thermo Fisher Scientific) was employed according to the manufacturer's instructions. On the following day, after washing, sections were incubated sequentially with Alexa Fluor 488‐ or Alexa Fluor 594‐conjugated secondary antibodies (Invitrogen) at room temperature for 1 h. Nuclei were counterstained with DAPI, and slides were mounted for imaging. Fluorescence images were acquired using a Nikon A1 laser scanning confocal microscope, and co‐localisation analysis was performed with ImageJ software.

### 2.9 Western Blot Analysis

Proteins were extracted from mouse kidney tissues and cultured cells using RIPA lysis buffer and then subjected to Western blot analysis as described previously. After blocking non‐specific binding with 5% BSA for 1 h, we incubated the membranes overnight at 4°C with primary antibodies against the following: collagen IA (1:1000; BA0325; Boster), Beclin1 (1:1000; T55092; Abmart), P62 (1:1000; T55546; Abmart), LC3B (1:1000; T55992; Abmart), total PI3K (1:1000; T40064; Abmart), phospho‐PI3K (1:1000; T40065; Abmart), total mTOR (1:1000; T55306; Abmart), phospho‐mTOR (1:1000; T56571; Abmart), TFEB (1:1000; TA7015; Abmart), total Akt (1:1000; 60203–2‐Ig; Proteintech), phospho‐Akt (1:1000; 66444–1‐Ig; Proteintech), fibronectin (1:1000; ab2413; Abcam), GDF15 (1:500; BS3818‐R; Bioss), and anti‐GFRAL (1:1000; ab214929; Abcam). Next, the membranes were exposed to the respective HRP‐conjugated secondary antibody (1:10000; EarthOx Life Sciences). Immunoreactive bands were subsequently detected and quantified using ECL Plus Western Blot Detection Reagents (Millipore Corp., Billerica, MA, USA) and Image J software, respectively.

### 2.10 Enzyme‐Linked Immunosorbent Assay (ELISA)

To quantify GDF15 levels, we performed ELISA assays on both cell culture supernatants and mouse serum samples. For HK2 cell culture supernatants, a human GDF15 ELISA kit (E‐EL‐H0080, Elabscience) was used according to the manufacturer's instructions. For serum samples from UUO and control mice, a mouse GDF15 ELISA kit (E‐EL‐M0604, Elabscience) was used. Absorbance was measured at 450 nm using a microplate reader, and all samples were run in duplicate.

### 2.11 Statistical Analysis

The data obtained are presented as means ± SD. To analyse normally distributed data, we performed a two‐tailed unpaired Student's *t*‐test for pairwise comparisons and a one‐way analysis of variance for comparisons involving multiple groups. For non‐normally distributed data, we employed the nonparametric Kruskal‐Wallis test and Mann–Whitney *U* test, utilising Prism software (version 9; GraphPad Software, San Diego, CA, USA).

## 3 Results

### 3.1 GDF15 Expression Is Decreased in TGF‐β‐Induced TIF In Vitro

**FIGURE 1:**
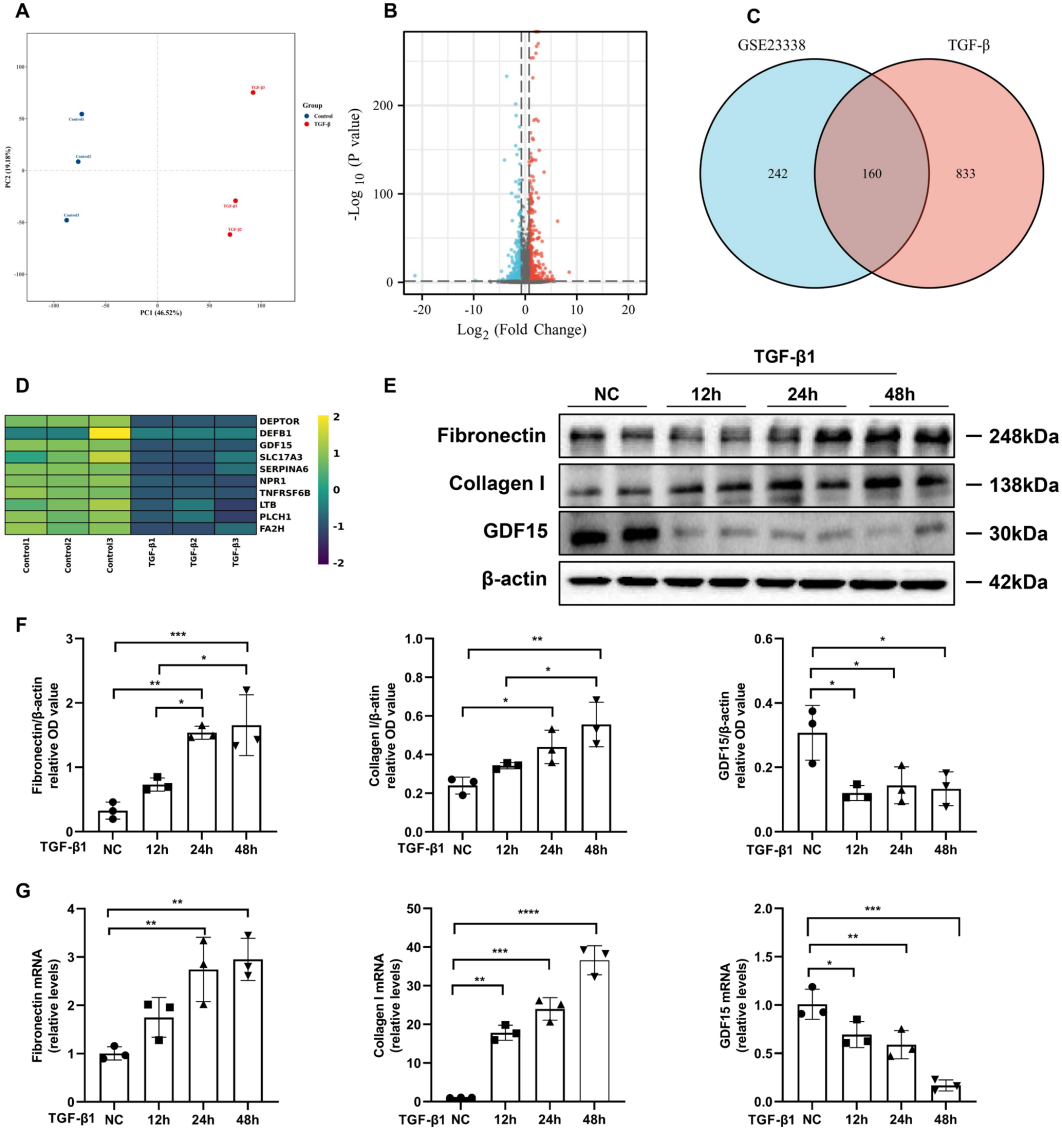
The relationship between GDF15 and tubulointerstitial fibrosis mediated by TGF β in HK2 cells. (A–D) Screening the differentially expressed genes related to TIF. (E, F) Western blot analysis and quantification of fibronectin, collagen I, and GDF15 in HK2 cells stimulated with 10 ng/mL of TGF‐β1 for 12, 24, and 48 h. (G) qRT‐PCR analysis of fibronectin, collagen I, and GDF15 levels in different groups as indicated. The relative expression levels of the indicated proteins were normalised to that of β‐actin (*n* = 3).*p < 0.05, **p < 0.01, ***p < 0.001, and ****p < 0.0001.

To elucidate differentially expressed genes in renal TIF, we performed transcriptome sequencing on TGF‐β1‐stimulated HK2 cells (Figure 1A,B) and leveraged transcriptomic data from the GSE23338 dataset, a TIF‐related dataset in the GEO database. Integration of these datasets revealed 160 significantly downregulated genes, with *GDF15* exhibiting the most prominent differential expression (Figure 1C,D). To further validate *GDF15* expression levels in renal TIF, we assessed its expression in TGF‐β1‐stimulated HK2 cells. Following TGF‐β1 treatment, the mRNA and protein expression of fibronectin and collagen exhibited a time‐dependent increase, peaking at 48 h, whereas GDF15 mRNA and protein expression decreased (Figure 1E–G). These findings indicate a downregulation of GDF15 expression in TGF‐β1‐stimulated HK2 cells, which is strongly correlated with TIF.

### 3.2 Downregulation of GDF15 Is Associated With the Progression of Renal Interstitial Fibrosis

**FIGURE 2:**
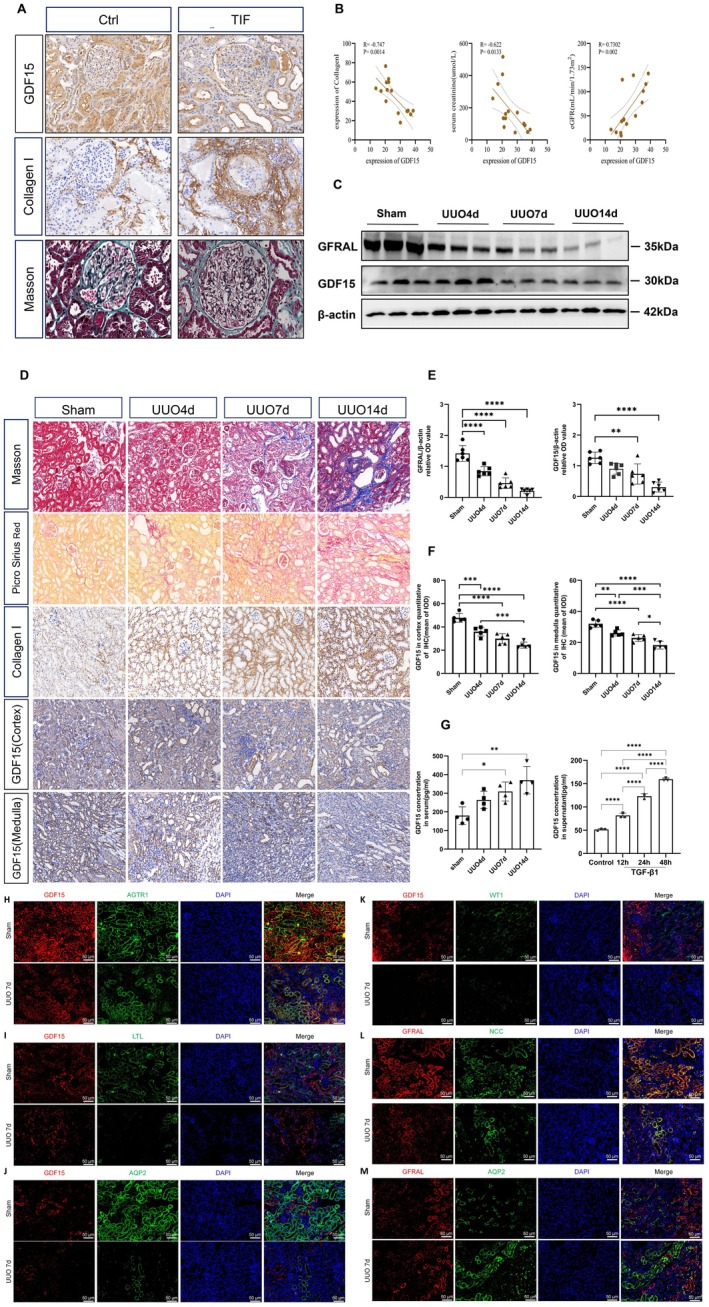
GDF15 expression in the fibrotic kidney of patients with CKD and UUO mice. (A) Immunohistochemical staining for Collagen I, GDF15 and representative images of Masson's trichrome staining in the kidneys of patients with CKD. (B) Correlation between Collagen I, serum creatinine, eGFR and the expression of renal GDF15 in patients with CKD. (C, E) Western blot analysis and quantification of the GDF15 and GFRAL levels in UUO mice. The relative expression levels of the indicated proteins were normalised to that of β‐actin. (D) Immunohistochemical staining for Collagen I, GDF15 and representative images of Masson's trichrome staining, Picrosirius red staining in the kidneys of UUO mice. (F) Quantitative analysis of immunohistochemistry for GDF15 in the cortex and medulla of UUO mice (*n* = 6). (G) Dynamic changes in serum GDF15 concentrations at different time points following UUO intervention in mice and GDF15 levels in the supernatant of TGF‐β1‐stimulated HK2 cells (*n* = 3). (H) Co‐staining of GDF15 with AGTR1: A distinct yellow signal in the merged image indicates that GDF15 is primarily localised in AGTR1‐positive tubular epithelial cells. (I) Co‐staining of GDF15 with the proximal tubule marker LTL: Absence of a yellow overlap in the merged image suggests weak GDF15 expression in proximal tubules. (J) Co‐staining of GDF15 with the collecting duct marker AQP2: No overlap signal is observed in the merged image, indicating minimal GDF15 expression in collecting ducts. (K) Co‐staining of GDF15 with WT1, a podocyte marker: The merged image shows no co‐localisation, suggesting that GDF15 is not expressed in glomerular podocytes. (L) Co‐staining of GFRAL with NCC: Strong overlap of red and green signals in the merged image indicates predominant localisation of GFRAL in the distal tubules. (M) Co‐staining of GFRAL with AQP2: No overlapping signal is observed in the merged image, demonstrating that GFRAL is not expressed in collecting ducts. Scale bars for all merged images = 50 μm (*n* = 6). *p < 0.05, **p < 0.01, ***p < 0.001, and ****p < 0.0001.

We examined GDF15 expression in kidney tissues from patients with CKD. A total of nine patients with varying degrees of renal interstitial fibrosis and either primary or secondary CKD were enrolled in this study, including six patients with IgA nephropathy (IgAN) and three with diabetic kidney disease (DKD). These cases were collectively classified as the TIF group. As a control group, six patients with minimal change disease (MCD) were included. Compared with the control group, patients in the TIF group were significantly older (47.11 ± 16.29 years vs. 27.83 ± 12.2 years, *p* = 0.0286). However, there were no significant differences between the groups in terms of sex distribution or body weight. We performed immunohistochemical staining to assess the expression and localisation of GDF15 and type I collagen in kidney tissues. The results showed a significant decrease in GDF15 expression in the TIF group compared with the control group (Figure 2A). Furthermore, we analysed the correlation between GDF15 expression and the degree of TIF, serum creatinine levels, and estimated glomerular filtration rate (eGFR). The analysis revealed that serum creatinine levels were elevated in the TIF group (178 vs. 77.5 μmol/L, *p* = 0.012), and type I collagen expression was significantly higher (56.32 ± 10.19 vs. 27.15 ± 4.72, *p* < 0.001). Additionally, the TIF group exhibited a marked reduction in eGFR (33.03 vs. 108.5 mL/min/1.73 m^2^, *p* = 0.0048), total cholesterol (6.04 vs. 9.58 ± 2.35 mmol/L, *p* = 0.0216), low‐density lipoprotein cholesterol (4.08 ± 2.53 vs. 7.25 ± 2.61 mmol/L, *p* = 0.0354), and GDF15 levels (20.33 ± 3.07 vs. 33.99 ± 3.87, *p* < 0.001). In contrast, there was no statistically significant difference in high‐density lipoprotein cholesterol levels between the two groups (Table 1). Further correlation analysis demonstrated that GDF15 expression negatively correlated with the severity of TIF (*R* = −0.747, *p* = 0.0014), suggesting that GDF15 levels declined as fibrosis progressed. Moreover, GDF15 expression in kidney tissues was inversely correlated with serum creatinine levels (*R* = −0.622, *p* = 0.0133) and positively correlated with eGFR (*R* = 0.7302, *p* = 0.002; Figure 2B). To further investigate the expression of GDF15 in TIF, we examined its localisation and expression levels in the kidneys of sham‐operated and UUO‐induced mice.

**TABLE 1:** Comparison of the clinical and laboratory characteristics between patient groups.

Variables	TIF group (*n* = 9)	Control group (*n* = 6)	*p* value
Clinical characteristics			
Age (years)	47.11 ± 16.29	27.83 ± 12.24	0.029
Male [*n* (%)]	6 (67.77)	4 (67.77)	1.000
Weight (kg)	66.46 ± 14.71	63.58 ± 11.12	0.577
Laboratory characteristics			
BUN (umol/L)	10.84 ± 6.19	7.62 ± 4.68	0.298
Serum creatitine (umol/L)	178 (134.5, 378)	77.5 (48.25, 118.3)	0.012
eGFR (mL/min/1.73 m^2^)	33.03 (15.21, 42.22)	108.5 (70.67, 135.0)	0.005
Hb (g/L)	124.1 ± 21.31	143.2 ± 25.29	0.139
CHO (mmol/L)	6.04 ± 2.70	9.58 ± 2.35	0.022
TG (mmol/L)	1.77 (1.27, 3.33)	1.67 (1.42, 2.64)	0.839
LDL‐c (mmol/L)	4.08 ± 2.53	7.25 ± 2.61	0.035
HDL‐c (mmol/L)	0.97 (0.90, 1.36)	1.79 (0.95, 2.45)	0.120
GDF15 (mean of IOD)	20.33 ± 3.07	33.99 ± 3.87	< 0.0001
Collagen I (mean of IOD)	56.32 ± 10.19	27.15 ± 4.72	< 0.0001

Abbreviations: CHO, total cholesterol; eGFR, estimated glomerular filtration rate; Hb, haemoglobin; HDL‐c, high‐density lipoprotein cholesterol; LDL‐c, low‐density lipoprotein cholesterol.

Histological analyses using Masson's trichrome and Picrosirius red staining revealed significant collagen deposition in the kidneys of UUO mice, which was further supported by immunohistochemical staining showing increased expression of collagen I (Figure 2D). These findings confirmed the successful establishment of the UUO‐induced TIF mouse model. Subsequent immunohistochemical analysis demonstrated a marked reduction in GDF15 expression in both the renal cortex and medulla of UUO mice (Figure 2D,F). Western blot analysis further confirmed that GDF15 and GFRAL expression levels progressively decreased with prolonged ureteral obstruction (Figure 2C,E). Interestingly, we also observed a time‐dependent increase in GDF15 levels in the supernatant of TGF‐β1‐stimulated HK2 cells and in the serum of UUO mice (Figure 2G), suggesting a potential role for GDF15 in systemic responses.

To further investigate the spatial localisation of GDF15 and its receptor GFRAL within the renal nephron, we performed immunofluorescence co‐staining using kidney tissues from both UUO and sham‐operated mice. GDF15 was predominantly expressed in AGTR1‐positive tubular epithelial cells, with strong yellow merged signals observed in the sham group, indicating clear co‐localisation, whereas GDF15 expression was much lower in UUO kidneys (Figure 2H). In contrast, weak or negligible co‐localisation was observed between GDF15 and LTL (proximal tubules; Figure 2I), and AQP2 (collecting ducts; Figure 2J), suggesting minimal expression in these nephron segments. Moreover, no co‐localisation was found between GDF15 and WT1, indicating the absence of GDF15 expression in podocytes (Figure 2K). GFRAL was mainly localised in NCC‐positive distal tubules with strong co‐localisation (Figure 2L), while no overlap was observed with AQP2 in collecting ducts (Figure 2M).

These observations indicate that GDF15 expression was significantly downregulated in the kidneys of UUO‐induced TIF mice, further underscoring the association between GDF15 expression and renal fibrosis.

### 3.3 Antifibrotic Effect of GDF15 on Cultured TGF‐β1‐Stimulated HK2s Cells

**FIGURE 3:**
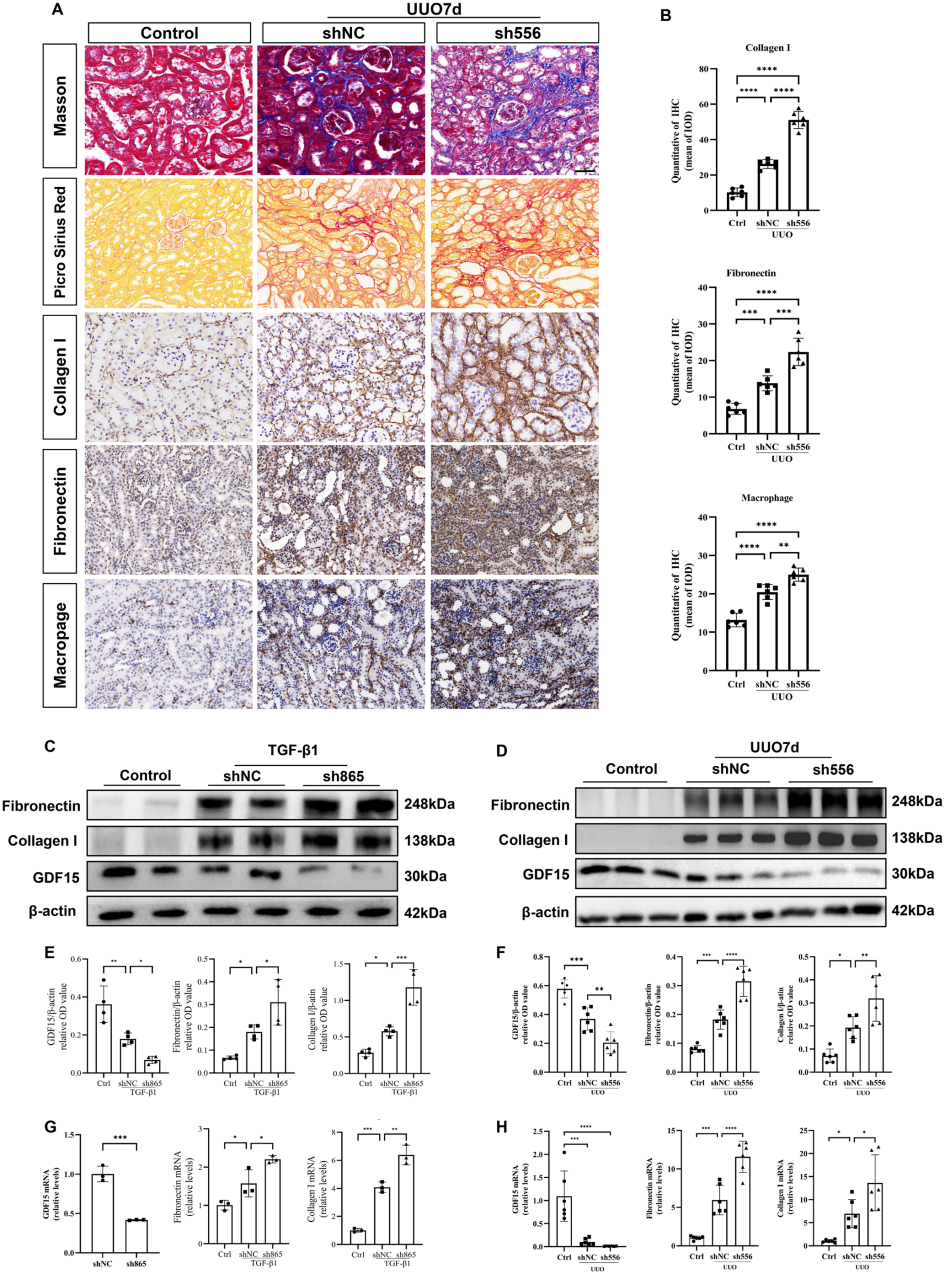
Downregulation of GDF15 enhances TGF β‐stimulated TIF in vitro and UUO‐induced TIF in vivo. (A) Representative images of Masson's trichrome staining, Picrosirius red staining and immunohistochemical staining for Collagen I, Fibronectin, and macrophage infiltration in the kidney homogenates from sham and UUO mice injected with shNC or sh556. (B) Quantitative analysis of immunohistochemistry for Collagen I, Fibronectin and macrophage in different groups as indicated. (C, E) Western blot analysis and quantification of the Collagen I, Fibronectin and GDF15 in vitro (*n* = 3). The relative expression levels of the indicated proteins were normalised to that of β‐actin. (G) qRT‐PCR analysis of fibronectin, collagen I, and GDF15 levels in vitro (*n* = 3). (D, F) Western blot analysis and quantification of the Collagen I, Fibronectin and GDF15 in vivo. The relative expression levels of the indicated proteins were normalised to that of β‐actin (*n* = 6). (H) qRT‐PCR analysis of fibronectin, collagen I, and GDF15 levels in vivo (*n* = 6). *p < 0.05, **p < 0.01, ***p < 0.001, and ****p < 0.0001.

**FIGURE 4:**
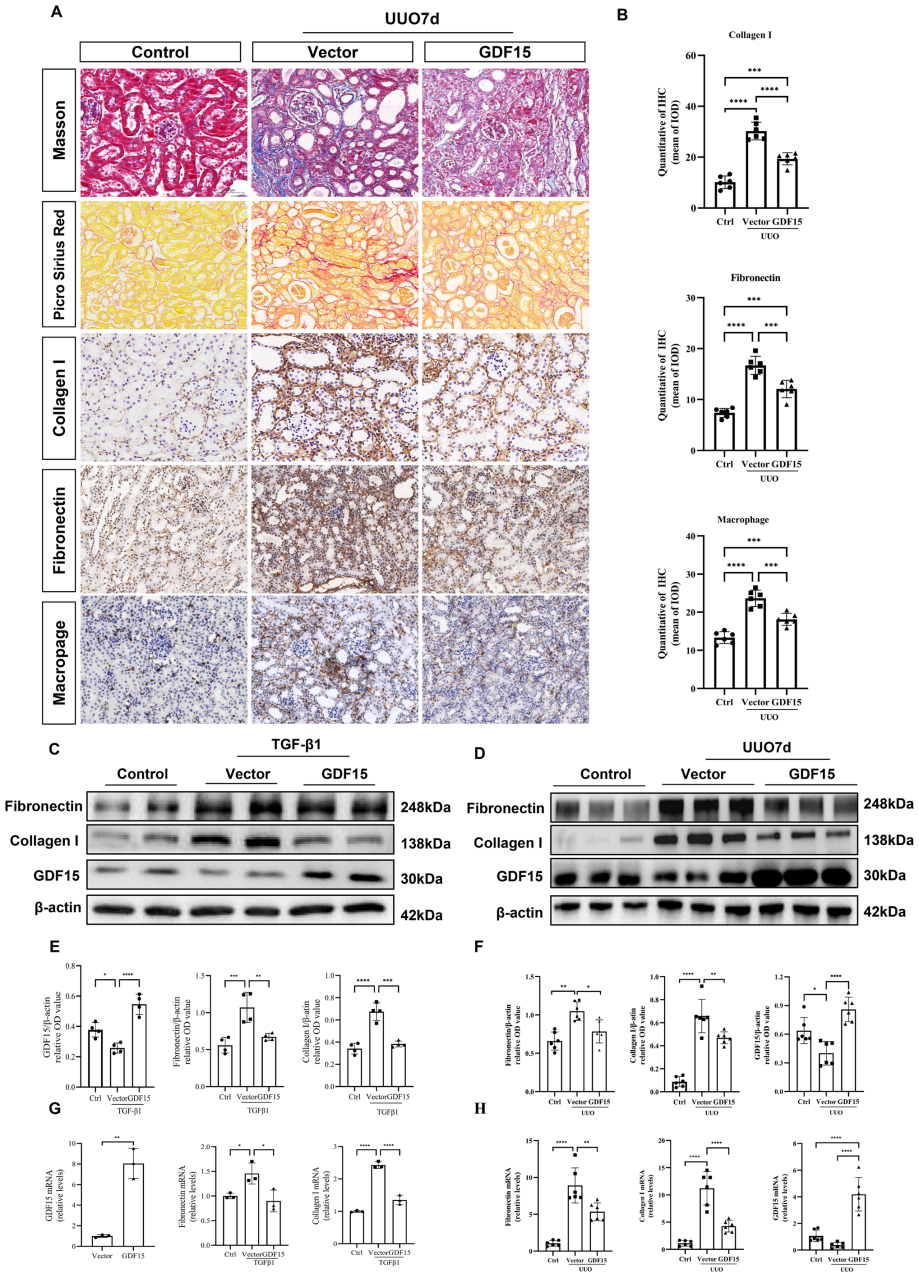
Overexpression of GDF15 ameliorated TGF‐β‐stimulated TIF in vitro and UUO‐induced TIF in vivo. (A) Representative images of Masson's trichrome staining, Picrosirius red staining and immunohistochemical staining for Collagen I, Fibronectin, and macrophage infiltration in the kidney homogenates from sham and UUO mice injected with empty vector or pGV362‐GDF15. (B) Quantitative analysis of immunohistochemistry for Collagen I, Fibronectin and GDF15 in different groups as indicated. (C, E) Western blot analysis and quantification of the Collagen I, Fibronectin and GDF15 in vitro (*n* = 3). The relative expression levels of the indicated proteins were normalised to that of β‐actin. (G) qRT‐PCR analysis of fibronectin, collagen I, and GDF15 levels in vitro (*n* = 3). (D, F) Western blot analysis and quantification of the Collagen I, Fibronectin and GDF15 in vivo. The relative expression levels of the indicated proteins were normalised to that of β‐actin (*n* = 6). (H) qRT‐PCR analysis of fibronectin, collagen I, and GDF15 levels in vivo (*n* = 6). *p < 0.05, **p < 0.01, ***p < 0.001, and ****p < 0.0001.

The relationship between GDF15 and TIF in cultured TGF‐β1‐stimulated HK2 cells was investigated using plasmids to modulate GDF15 expression, both downregulating and upregulating it. Transfection with GDF15 shRNA (sh556) resulted in a substantial reduction of approximately 60% in GDF15 mRNA levels (Figure 3G), while cells transfected with pGV362‐GDF15 exhibited an approximate eight‐fold increase in GDF15 mRNA levels compared to cells transfected with the vector control (Figure 4G). To determine whether GDF15 was associated with ECM degradation, we treated HK2 cells with or without TGF‐β1 in the presence of GDF15‐inhibiting or GDF15‐overexpressing plasmids. Notably, GDF15 shRNA upregulated the mRNA and protein expression of fibronectin and collagen I in TGF‐β1‐stimulated HK2 cells (Figure 3C,E,G). Conversely, GDF15 overexpression decreased the mRNA and protein expression of fibronectin and collagen I (Figure 4C,E,G). Collectively, these findings indicate that silencing GDF15 exacerbates ECM deposition, while upregulating GDF15 expression reduces ECM deposition in TGF‐β1‐stimulated HK2 cells.

### 3.4 Reno‐Protective Effect of GDF15 in a UUO‐Induced TIF Mouse Model

The diminished expression of GDF15 in the kidneys of UUO‐induced TIF mice and its correlation with TIF implied a potential role for GDF15 in TIF pathogenesis. To investigate this hypothesis, we upregulated GDF15 expression in mouse kidneys by microbubble‐mediated delivery of a GDF15 plasmid 2 days before surgery. Western blot analysis validated the upregulation of GDF15 expression in kidney homogenates from the pGV362‐GDF15 group when compared to the UUO group (Figure 4D). GDF15 overexpression mitigated the upregulation of fibronectin and collagen I expression induced by UUO. Masson's trichrome and Picrosirius red staining revealed that GDF15 overexpression ameliorated collagen deposition (Figure 4A), while immunohistochemical staining, Western blot and qRT‐PCR demonstrated the reversal of fibronectin and collagen I upregulation, along with reduced macrophage infiltration caused by UUO (Figure 4A,B,D,F,H). Conversely, the downregulation of GDF15 expression via the introduction of GDF15 shRNA (sh556) into the kidneys led to reduced GDF15 expression, upregulation of fibronectin and collagen I owing to UUO, accelerated collagen deposition, and increased macrophage infiltration caused by UUO (Figure 3A,B,D,F,H). Overall, these findings suggest that silencing GDF15 exacerbates TIF and macrophage infiltration, while upregulating GDF15 expression diminishes TIF and macrophage infiltration in the UUO‐induced TIF mouse model.

### 3.5 GDF15 Promoted Autophagy and Lysosome Biogenesis by Inhibiting the PI3K/Akt/mTOR Pathway

**FIGURE 5:**
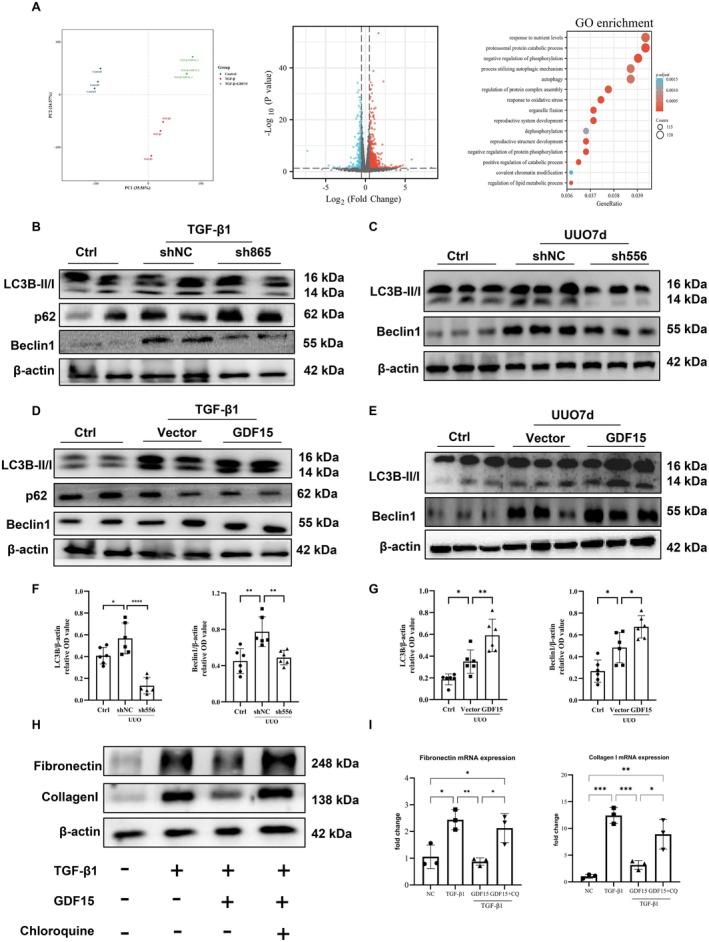
GDF15 ameliorates renal fibrosis through enhancing autophagy. (A) Bioinformatics predicts the mechanism of GDF15: PCA; Differentially clustered volcano plot; Top 15 of GO enrichment. (B) Western blot analysis of the LC3B, Beclin1 and p62 in TGF‐β‐stimulated TIF with transfection of shNC or sh865 (*n* = 3). (C, F) Western blot analysis and quantification of the LC3B, Beclin1 in UUO‐induced TIF with injecting shNC or sh556 (*n* = 6). (D) Western blot analysis of the LC3B, Beclin1 and p62 in TGF‐β‐stimulated TIF with transfection of empty vector or pGV362‐GDF15 (*n* = 3). (E, G) Western blot analysis and quantification of the LC3B, Beclin1 in UUO‐induced TIF with injecting empty vector or pGV362‐GDF15 (*n* = 6). (H) Western blot analysis the Collagen I and Fibronectin in TGF‐β‐stimulated TIF with transfection of empty vector, pGV362‐GDF15 and chloroquine treatment (*n* = 3). (I) qRT‐PCR analysis of fibronectin and collagen I levels in different groups as indicated. *p < 0.05, **p < 0.01, ***p < 0.001, and ****p < 0.0001.

**FIGURE 6:**
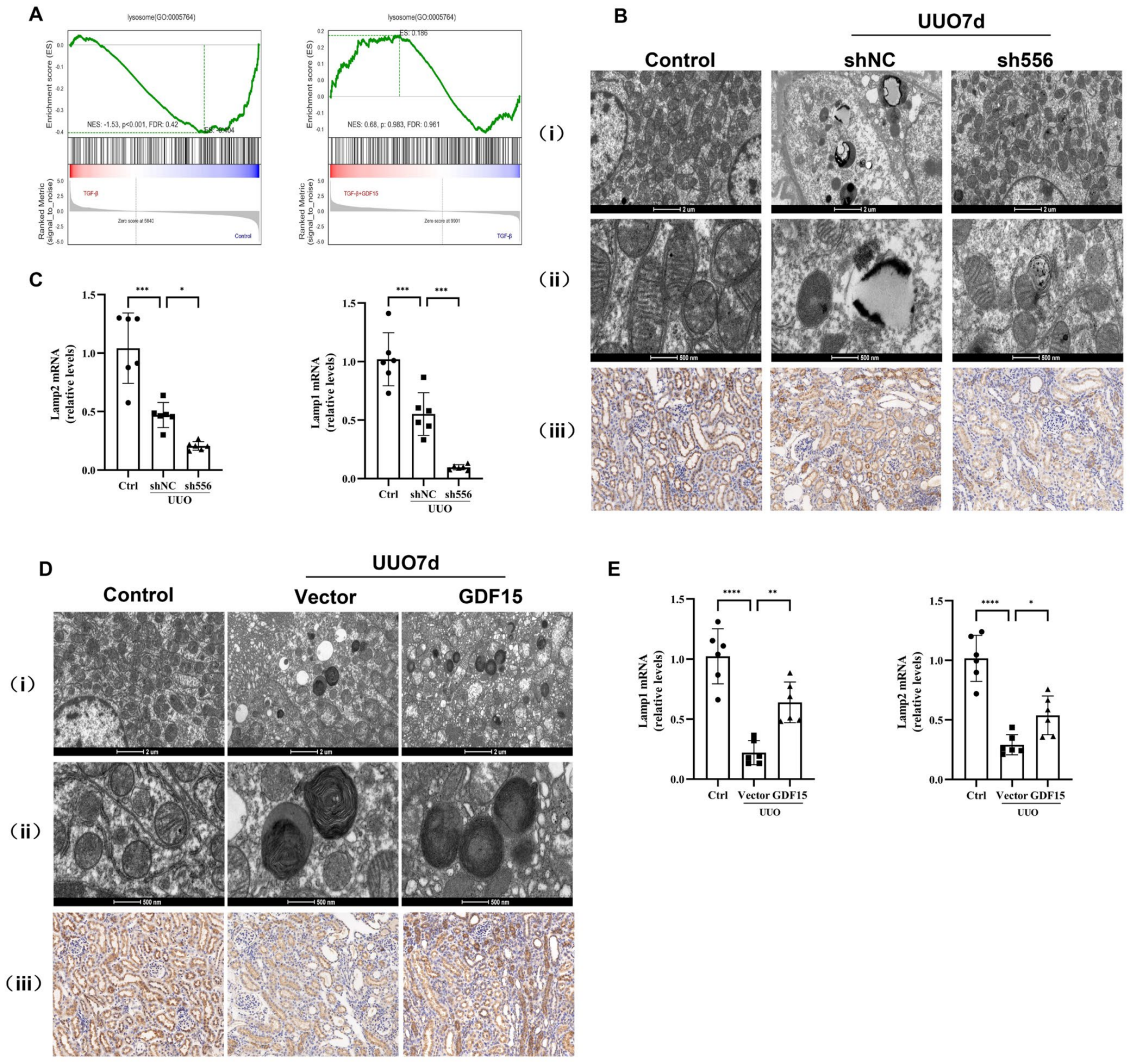
GDF15 ameliorates renal fibrosis through improving lysosomal neogenesis. (A) GSEA enrichment. (B) Downregulation of kidney GDF15 in the UUO model, electron microscopy detected the number of lysosomes (i, ii), the scale bars in the upper‐row images represent 2 μm, and those in the lower‐row images represent 500 nm and immunohistochemical staining analysis of the LAMP (iii). (C) qRT‐PCR analysis of Lamp1 and Lamp2 in different groups as indicated. (D) Overexpression of kidney GDF15 in the UUO model, electron microscopy detected the number of lysosomes (i, ii), the scale bars in the upper‐row images represent 2 μm, and those in the lower‐row images represent 500 nm and immunohistochemical staining analysis of the LAMP (iii). (E) qRT‐PCR analysis of Lamp1 and Lamp2 in different groups as indicated (*n* = 3). *p < 0.05, **p < 0.01, ***p < 0.001, and ****p < 0.0001.

To further investigate the underlying mechanism of GDF15's protective effect on renal interstitial fibrosis, we conducted bioinformatics analysis and RNA transcriptome sequencing to identify downstream target genes of GDF15 after upregulating its expression. GO enrichment analysis and GSEA revealed significant enrichment associated with autophagy and lysosomes (Figures 5A and 6A). To validate GDF15's protective effect via autophagy, we assessed changes in levels of autophagy‐related proteins using Western blot. The results demonstrated that the downregulation of GDF15 expression resulted in significantly decreased expression of LC3 and Beclin1, indicating reduced autophagy levels compared to the ShNC group (Figure 5B,C,F). Conversely, the GDF15 group displayed significantly increased expression of LC3 and Beclin1, along with reduced accumulation of the autophagy substrate p62 compared to the vector group (Figure 5D,E,G). These findings validate that GDF15 induces autophagy in renal tubular epithelial cells, aligning with the results observed in the in vivo study.

**FIGURE 7:**
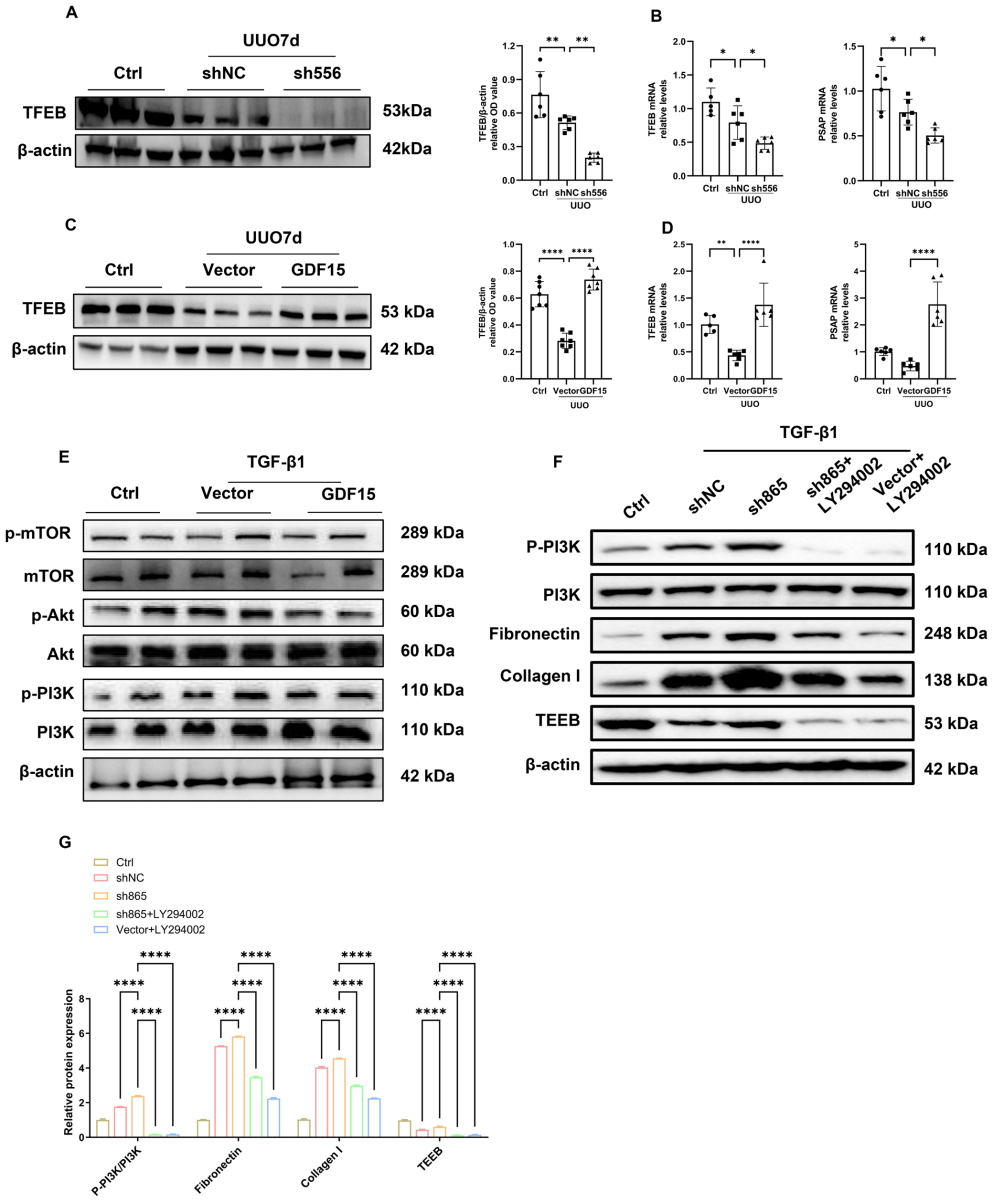
GDF15 promoted autophagy and lysosome biogenesis by inhibiting the PI3K/Akt/mTOR pathway. (A) Downregulation of kidney GDF15 in the UUO model, Western blot analysis and quantification of TFEB. The relative expression levels of the indicated proteins were normalised to that of β‐actin. (B) qRT‐PCR analysis of TFEB and PASP in different groups as indicated. (C) Overexpression of kidney GDF15 in the UUO model, Western blot analysis and quantification of TFEB. The relative expression levels of the indicated proteins were normalised to that of β‐actin. (D) qRT‐PCR analysis of TFEB and PASP in different groups as indicated. (E) Western blot analysis of p‐mTOR, mTOR, p‐Akt, Akt, p‐PI3K, and PI3K levels in TGF β‐stimulated TIF with transfection of empty vector or pGV362‐GDF15. (F, G) Effects of GDF15 silencing and PI3K inhibition on the PI3K signalling pathway and the expression of fibrosis‐ and autophagy‐related proteins. HK‐2 cells were transfected with shGDF15 (sh865) or control vector (shNC) for 48 h, followed by treatment with the PI3K inhibitor LY294002 (final concentration 20 μM) for 24 h. Western blot and quantification was performed to detect the expression levels of p‐PI3K, PI3K, fibronectin, collagen I, TFEB, and the internal control β‐actin. All data were normalised to β‐actin (*n* = 3). *p < 0.05, **p < 0.01, ***p < 0.001, and ****p < 0.0001.

Next, we further validated whether GDF15 ameliorates TGF‐β1‐induced TIF by activating autophagy. Hydroxychloroquine was employed to inhibit autophagy, and the effect of GDF15 on TGF‐β1‐induced TIF was observed. The results demonstrated that, compared to the GDF15 overexpression group, the protein and mRNA expression of fibronectin and collagen I significantly increased (Figure 5H,I), suggesting that GDF15 alleviates TGF‐β1‐induced ECM deposition by activating autophagy. Furthermore, we examined the number of lysosomes in UUO‐induced TIF mice using transmission electron microscopy. Autophagosomes with multilayer membranes were observed in the sh556 group. Additionally, the number of lysosomes in the sh556 group was lower compared to the shNC group (Figure 6B). However, upregulating GDF15 expression led to a further increase in lysosomal expression, characterised by an expansion of the inner low electron density area and a thinning of the multilayer membrane structure (Figure 6D). Further analysis revealed that after upregulating GDF15 expression, the mRNA level of PSAP significantly decreased, while downregulating GDF15, the mRNA levels of Lamp1, Lamp2, TFEB, and PSAP significantly increased (Figures 6C,E and 7A–D). Immunohistochemistry and Western blot further validated these changes, indicating that GDF15 promoted lysosomal function.

Moreover, mechanistic studies showed that upregulation of GDF15 inhibited the phosphorylation of the PI3K/Akt/mTOR pathway (Figure 7E), whereas silencing GDF15 produced the opposite effect (Figure 7F,G). Concurrently, the reduction in phosphorylated PI3K was further enhanced in the LY294002‐treated group, indicating that suppression of PI3K phosphorylation plays a critical role in GDF15‐mediated autophagy and fibrotic responses. These findings suggest that GDF15 may alleviate TIF by activating autophagy through inhibition of the PI3K/Akt/mTOR pathway.

### 3.6 GDF15 Mediates Autophagy Activation and Anti‐Fibrotic Effects via GFRAL

**FIGURE 8:**
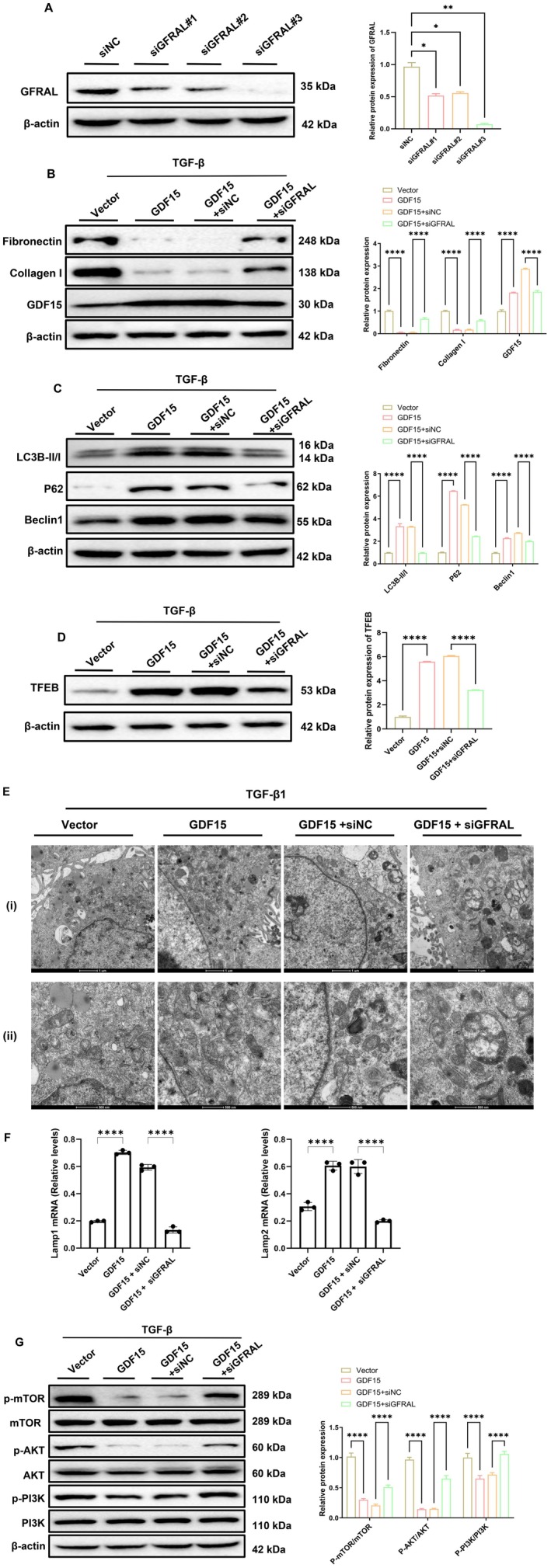
GDF15 mediates autophagy activation and anti‐fibrotic effects through GFRAL. (A) Western blot analysis and quantification of GFRAL protein expression in HK2 cells following siRNA‐mediated knockdown. siNC served as the negative control, and siGFRAL#1, #2, and #3 represent different interference sequences. (B) Western blot analysis and quantification of fibronectin, collagen I, and GDF15 protein expression in various treatment groups (Vector, GDF15, GDF15 + siNC, GDF15 + siGFRAL) under TGF‐β stimulation. β‐actin was used as the loading control. (C) Western blot analysis and quantification of LC3B, P62, and Beclin1 protein levels in each group. (D) Western blot analysis and quantification of TFEB protein expression in each group. (E) Transmission electron microscopy was used to observe the structural changes of lysosomes in HK2 cells from each group. The scale bars in the upper‐row images represent 1 μm, and those in the lower‐row images represent 500 nm. (F) qRT‐PCR analysis of Lamp1 and Lamp2 in different groups as indicated. (G) Western blot analysis and quantification of PI3K/AKT/mTOR signalling pathway proteins, including p‐mTOR, mTOR, p‐AKT, AKT, p‐PI3K, and PI3K. β‐actin was used as the loading control (*n* = 3).*p < 0.05, **p < 0.01, ***p < 0.001, and ****p < 0.0001.

To further determine whether the autophagy‐activating and anti‐fibrotic effects of GDF15 under TGF‐β stimulation depend on its canonical receptor GFRAL, we constructed a GDF15‐overexpressing model in HK2 cells and silenced GFRAL expression using siRNA. Among three candidates, siGFRAL‐1, siGFRAL‐2, and siGFRAL‐3, siGFRAL‐3 exhibited the highest silencing efficiency and was selected for subsequent experiments (Figure 8A). Under TGF‐β stimulation, GDF15 overexpression significantly suppressed the expression of fibrotic markers fibronectin and collagen I (Figure 8B). This inhibitory effect was partially reversed upon GFRAL knockdown, suggesting that GFRAL plays a critical role in GDF15‐mediated anti‐fibrotic activity.

Further analysis of autophagy‐related proteins revealed that GDF15 significantly increased LC3B‐II/I and Beclin1 levels, while decreasing the autophagy substrate p62, indicating activation of the autophagic pathway (Figure 8C). These effects were substantially attenuated following silencing of GFRAL. Concurrently, GDF15 upregulated the expression of TFEB, a key regulator of lysosomal biogenesis, and this effect was also suppressed upon GFRAL knockdown (Figure 8D). Transmission electron microscopy confirmed enhanced lysosome formation in the GDF15 group, while siGFRAL intervention reduced lysosome numbers, further supporting GFRAL dependency (Figure 8E). To further elucidate the mechanism of GDF15/GFRAL action, we examined the activity of the PI3K/AKT/mTOR signalling axis. The results showed that GDF15 suppressed the phosphorylation of PI3K, AKT, and mTOR, indicating inhibition of the pathway (Figure 8G). This suppression was reversed following GFRAL knockdown, suggesting that GDF15 activates autophagy by inhibiting the PI3K/AKT/mTOR pathway via GFRAL.

Quantitative analyses further supported these findings. RT‐qPCR revealed that GDF15 upregulated the transcription of Lamp1 and Lamp2, while this effect was significantly diminished in the siGFRAL group (Figure 8F). Collectively, these results indicate that GDF15 exerts anti‐fibrotic and autophagy‐promoting effects through its canonical receptor, GFRAL, and that activation of autophagy is mediated by the inhibition of the PI3K/AKT/mTOR pathway.

## 4 Discussion

This study demonstrated that GDF15 expression is decreased in fibrotic kidneys in both patients with CKD and a murine model of UUO. This downregulation is correlated with progressive renal fibrosis and a decline in eGFR in patients with CKD. Using RNA‐seq, immunohistochemical staining, and Western blot analysis, we identified GDF15 expression in both the cortical and medullary regions of kidney tissues. Importantly, our findings indicate that renal fibrosis exacerbation is associated with decreased expression of GDF15. Collectively, these observations suggest that GDF15 holds promise as a potential therapeutic target for mitigating renal fibrosis. We further substantiated this finding by silencing or overexpressing GDF15 in vitro and in vivo models of renal fibrosis.

Mechanistically, GDF15 ameliorates renal fibrosis by enhancing autophagy through the PI3K/Akt/mTOR pathway. This is substantiated by evidence showing that the overexpression of GDF15, both in vitro and in vivo, leads to increased lysosome production and activation of autophagy. Conversely, the downregulation of GDF15 results in a reduced number of newly formed lysosomes and inhibition of autophagy.

Additional investigations indicate that the use of hydroxychloroquine to inhibit autophagy counteracts the ameliorative effect of GDF15 overexpression on renal interstitial fibrosis. Moreover, mechanistic studies elucidate that GDF15 overexpression inhibits the PI3K/Akt/mTOR pathway. GDF15, a member of the TGF‐β superfamily, functions as an autocrine regulatory molecule in macrophages, inhibiting TNF‐α production in lipopolysaccharide‐induced macrophages. Hence, it is aptly named macrophage inhibitory cytokine‐1. GDF15 is initially synthesised in the cytoplasm as pro‐GDF15, which subsequently undergoes cleavage to form a mature dimeric structure before being secreted into the bloodstream [24, 25]. Both pro‐GDF15 within cells and mature GDF15 in circulation exert regulatory control over cell growth, differentiation, and inflammation, thereby playing vital roles in physiological processes. Our study advances this understanding by underscoring that GDF15 expression decreases in the kidneys of mice with UUO‐induced TIF and TGF‐β1‐stimulated HK2 cells. This decrease indicates a correlation with renal fibrosis. Moreover, our findings demonstrate that inhibiting GDF15 expression exacerbates ECM accumulation, while upregulating GDF15 diminishes renal fibrosis. Previous research on idiopathic pulmonary fibrosis, utilising single‐cell RNA sequencing, has revealed that epithelial cells are a major source of GDF15. Moreover, elevated GDF15 levels in the bloodstream exhibit a significant correlation with disease severity and survival rates, implying GDF15's potential utility as a biomarker for epithelial stress [26]. Furthermore, studies have demonstrated the potential of GDF15 to mitigate fibrotic changes in the lungs by disrupting the TGF‐β/Smad pathway, thereby inhibiting the growth and activation of lung fibroblasts [27]. In mouse models of nonalcoholic steatohepatitis and myocardial infarction, the knockout of GDF15 can result in increased fibrosis, leading to higher mortality. This mechanism may be associated with GDF15's ability to block chemokine‐triggered leukocyte activation, inhibit neutrophil recruitment, and reduce collagen deposition [28, 29, 30, 31]. Our study further substantiates this by revealing that knocking down GDF15 expression significantly increases macrophage infiltration in the renal tubulointerstitium. Conversely, upregulating GDF15 expression significantly reduces such infiltration, suggesting a role for GDF15 in kidney function, potentially through the reduction of tubulointerstitial inflammation. This finding is consistent with that of a previous study that demonstrated GDF15's reno‐protective effect by preserving Klotho storage in CKD and AKI models [32].

In previous studies, GDF15 has been demonstrated to regulate renal ECM production and enhance the proliferation of renal tubular epithelial cells through an atypical mitogen‐activated protein kinase signalling pathway [20]. It may also contribute to maintaining tubular integrity by promoting tubular repair [19]. Intrarenal expression of GDF15 can be induced by renal injury via TNF‐ and p53‐dependent and independent mechanisms [15]. This induction triggers protective responses, such as the activation and proliferation of renal tubular epithelial cells, the inhibition of ECM protein accumulation, and the activation of inflammatory cells. These findings are consistent with the overexpression of GDF15, reduced deposition of ECM, and reduction of macrophage infiltration observed in our study. Additionally, we found that overexpression of GDF15 enhances autophagy and lysosomal enrichment. Autophagy, a highly conserved protein degradation system within cells, plays a crucial role in modulating energy metabolism, intracellular quality control, and cell development and differentiation through the degradation and recycling process.

It influences a wide range of cellular processes, including cell death, inflammation, and immune response, thereby serving as a potential modulator of both adaptive and maladaptive roles in disease pathogenesis [33]. Recent studies have elucidated that TECs induce autophagy in response to various injuries, such as exposure to renal toxic substances [34, 35, 36, 37], ureteral obstruction [38], and renal ischemia–reperfusion [39]. The robustness of their protective autophagic responses determines the proliferative ability of TECs post‐injury [40]. We speculate that GDF15 fosters renal protection by promoting autophagy in renal tubular cells. Further investigations reveal that the upregulation of GDF15 results in an increase in the expression of autophagy‐related proteins and a decrease in the expression of autophagy substrates. Conversely, inhibiting autophagy through the use of the autophagy‐specific inhibitor chloroquine impedes tubular ECM deposition. Additionally, we found that the expression of TFEB, the principal lysosomal nascent transcription factor, increased following GDF15 upregulation. Immunohistochemical staining revealed elevated expression of the nascent lysosomal membrane protein LAMP1, and electron microscopy indicated an upsurge in the number of lysosomes following GDF15 upregulation. In summary, we propose that GDF15 plays an anti‐renal fibrotic role by activating protective autophagy in renal tubular epithelial cells and inducing lysosome neogenesis.

Despite the protective role of GDF15 in renal interstitial fibrosis demonstrated in this study, several limitations remain. First, the control group was relatively younger, which may have influenced the expression levels of GDF15. Future studies should include age‐matched samples and larger cohorts to enable multivariate analyses. Second, although we employed LY294002 to verify the role of the PI3K/Akt/mTOR pathway in GDF15‐mediated autophagy and anti‐fibrotic effects, further interventions targeting Akt or mTOR specifically were not conducted. Additionally, the antibodies used for tissue analysis failed to distinguish between pro‐GDF15 and mature GDF15. Thus, the detected signals primarily reflect total GDF15 levels in tissues, whereas ELISA in serum predominantly measures the mature form. The observed elevation of serum GDF15 in the UUO model suggests a potential involvement in systemic responses; however, the relative contributions of local versus systemic sources remain to be clarified. Regarding receptor mechanisms, our study validated only the function of GFRAL, a high‐affinity receptor supported by previous literature [41], and did not experimentally assess the role of ErbB2. Future investigations are warranted to explore its potential involvement. More definitive mechanistic evidence will require in vivo validation using transgenic or conditional knockout mouse models with tubule‐specific manipulation of GDF15.

In summary, our study demonstrated that GDF15 expression is decreased in renal TIF and is negatively correlated with the degree of renal TIF. Additional experiments, both in vivo and in vitro models, revealed that knocking down the expression of GDF15 significantly exacerbates ECM deposition and macrophage infiltration in renal TIF, whereas overexpression of GDF15 is associated with increased TIF and macrophage infiltration. These findings suggest that GDF15 plays a protective role in TIF. Further analysis revealed that GDF15 promotes autophagy and lysosomal function, thereby ameliorating ECM deposition, which may be one of the mechanisms by which GDF15 exerts its renoprotective effect.

## Author Contributions

**Youqi Li:** conceptualization (lead), data curation (lead), formal analysis (lead), funding acquisition (equal), investigation (lead), methodology (lead), project administration (equal), resources (lead), software (lead). **Jinge Gu:** data curation (equal), formal analysis (equal). **Danping Tao:** investigation (equal). **Haoyang Wu:** methodology (supporting). **Chen Yang:** investigation (supporting), methodology (supporting). **Yongjun Shi:** project administration (supporting). **Chengwen Huang:** resources (supporting). **Boxun Luo:** resources (equal), supervision (equal), writing – review and editing (equal). **Jun Zhang:** supervision (equal).

## Conflicts of Interest

The authors declare no conflicts of interest.

## Supporting information


**Table S1:** jcmm70951‐sup‐0001‐Table S1‐S2.docx.
**Table S2:** jcmm70951‐sup‐0001‐Table S1‐S2.docx.

## Data Availability

The data that support the findings of this study are openly available inScience Data Bank https://www.scidb.cn at https://www.scidb.cn/s/fau26r.
