# Supplementary material for: Novel Roles of GDF15 in Alleviating Renal Fibrosis: Promoting Autophagy and Lysosome Biogenesis via Inhibition of the PI3K/Akt/mTOR Pathway
Source: J Cell Mol Med. 2025 Dec 31;30(1):e70951. doi: 10.1111/jcmm.70951 (PMC12755054; doi:10.1111/jcmm.70951)
Supplement: Supplementary file 1 — Table S1: jcmm70951‐sup‐0001‐Table S1‐S2.docx. Table S2: jcmm70951‐sup‐0001‐Table S1‐S2.docx. [file JCMM-30-e70951-s001.docx]

**Table S1. siRNA sequences.**

| **Name** | **Strand** | **Sequence** |
| --- | --- | --- |
| siRGFRAL1 | Sense | 5'-GAGTGGCGGAAGTTGATAA-3' |
|  | Antisense | 5'-UCAACTTCCGCCACTCGU-3' |
| siRGFRAL2 | Sense | 5'-GCCCUCGGGCUUGAGAAUA-3' |
|  | Antisense | 5'-UUAUUCUCAAGCCCGAGGC-3' |
| siRGFRAL3 | Sense | 5'-GGAAGTTGATAAGTGGGAT-3' |
|  | Antisense | 5'-AUCCCACUUCUAACUCCUU-3' |
| siNC | Sense | 5'-UUCUCCGAACGUGUCACGUTT-3' |
|  | Antisense | 5'-ACGUGACACGUUCGGAGAA TT-3' |

**Table S2. Primers used to quantify mRNA expression.**

| **Gene** | **FP sequence (5′–3′)** | **RP sequence (5′–3′)** |
| --- | --- | --- |
| Human collagen I | ATCACCAGGGAGGAATTTCCGT | CACCAGGAGCACGAGGTTTTC |
| Human fibronectin | CCATTCCTGCGGCAACACCAATC | CCCGAGAGAGATACGCAGGTGC |
| Human GDF15 | CAACCAGAGCTGGGAAGATTCG | GTGATGATGTCTTCATTTCCAGCAC |
| Human GAPDH | GAAGGTGAAGGTCGGAGTCA | CATGGGTGGAATCATATTGGAA |
| Human LAMP1 | CGTGTCACGAAGGCGTTTTCAG | CTGTTCTCGTCCAGCAGACACT |
| Human LAMP2 | GGCAATGATACTTGTCTGCTGGC | GTAGAGCAGTGTGAGAACGGCA |
| Mice collagen I | CCTTCTGGTCCTCCTGGTCCTCC | AGCCTCGGTGTCCCTTCATTCC |
| Mice fibronectin-1 | CCATTCCTGCGGCAACCAATC | GAGAGCTCCGGGCATTCCCACT |
| Mice GDF15 | CAACCAGAGCTGGGAAGATTCG | CCCGAGAGAGATACGCAGGTGC |
| Mice fibronectin-2 | GATGTCCGAACAGCTATTTACCA | CCTTGGGACTTCAGCCACT |
| Mice GDF15-2 | CGCTGCTGTGTCATCTTG | CAAGCCAGTCCTGGTTCTCA |
| Mice GAPDH | AATGGTGAAGGTCGGTGTGA | TGAAGGGGTCGTTGATGGC |
